# Systematic study of the dynamics and half-lives of newly synthesized proteins in human cells†
†Electronic supplementary information (ESI) available. See DOI: 10.1039/c5sc03826j


**DOI:** 10.1039/c5sc03826j

**Published:** 2015-11-16

**Authors:** Weixuan Chen, Johanna M. Smeekens, Ronghu Wu

**Affiliations:** a School of Chemistry and Biochemistry and the Petit Institute for Bioengineering and Bioscience , Georgia Institute of Technology , Atlanta , Georgia 30332 , USA . Email: Ronghu.Wu@chemistry.gatech.edu ; Fax: +1-404-894-7452 ; Tel: +1-404-385-1515

## Abstract

Protein dynamics are essential in regulating nearly every cellular event, and aberrant proteostasis is the source of many diseases. It is extraordinarily difficult to globally study protein dynamics and accurately measure their half-lives. Here we have developed a chemical proteomics method integrating protein labeling, click chemistry and multiplexed proteomics, which overcomes current challenges with existing methods. Labeling with both azidohomoalanine (AHA) and heavy lysine allows us to selectively enrich newly synthesized proteins, clearly distinguish them from existing proteins, and reduce the impact of heavy amino acid recycling. Moreover, multiplexed proteomics enables us to quantify proteins at multiple time points simultaneously, thus increasing the accuracy of measuring protein abundance changes and their half-lives. Systematic investigation of newly synthesized protein dynamics will provide insight into proteostasis and the molecular mechanisms of disease.

## Abstract 2

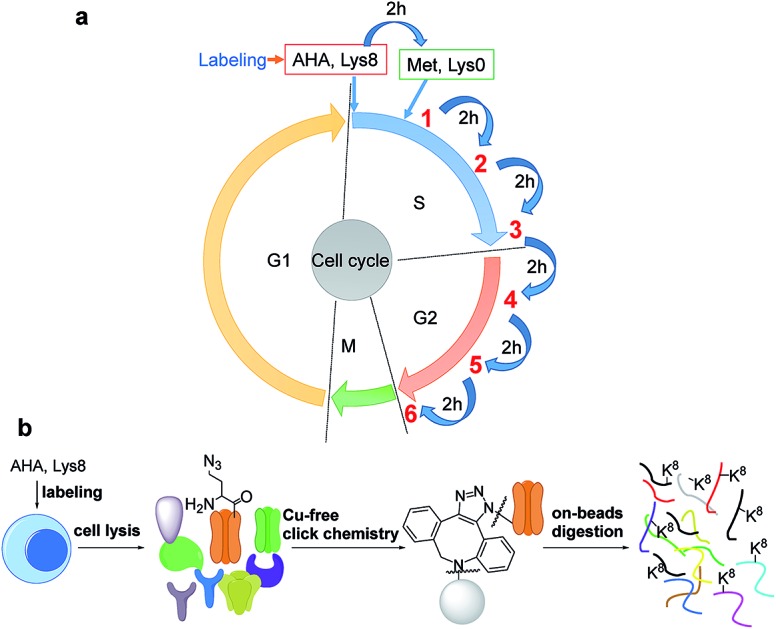
A novel chemical proteomics method was developed to specifically identify newly synthesized proteins and measure their half-lives.

## Introduction

Protein homeostasis (proteostasis) is critical for nearly every cellular event. The proper maintenance of proteostasis allows cells to grow and proliferate, respond to environmental cues and nutrient supplies, and defend cells from being attacked by pathogens.^1^,^2^ Aberrant proteostasis is the source of many diseases, including neurodegenerative, autoinflammatory and cardiovascular diseases.^3^–^6^ The maintenance of cellular proteostasis requires a balance of protein synthesis, trafficking and degradation. The degradation of newly synthesized proteins is an extremely important component of proteostasis because excess proteins are not only a burden for cells, but also a waste of cell energy.

Protein dynamics have been a long-standing interest in the biological and biomedical research fields. Early protein dynamics studies relied on the incorporation of radioactive elements into newly synthesized proteins, and the decay in radioactivity was measured over time to study the protein degradation.^7^,^8^ Although this method typically obtains information regarding overall protein degradation, the use of radioactive elements could lead to health problems. In order to analyze individual proteins, antibodies are required, which make large-scale analysis difficult. Fluorescence-based methods have also been developed to detect newly synthesized proteins and measure their half-lives.^9^,^10^ However, they typically require proteins to be tagged with a fluorescence probe and then measured individually, making comprehensive protein analysis time-consuming. Mass spectrometry (MS) combined with pulse-chase stable isotope labeling by amino acids in cell culture (SILAC) is currently a very popular method, and has been extensively applied to investigate protein turnover and degradation.^11^,^12^ However, there are several challenges with this method. First, it cannot selectively enrich targeted and labeled proteins; therefore, low abundance proteins or proteins with a high degradation rate could be missed for detection at later chase points, resulting in inaccurate abundance measurements. In addition, many existing proteins may interfere with peptide quantification during MS analysis. Second, a portion of heavy amino acids are always recycled by the cell during the chase step. Although the recycling effects are often ignored, it may have dramatic effects on the accurate quantification of protein degradation.^13^ Ideally, the method would be high-throughput and selective for newly synthesized proteins, so that it can be used to accurately measure protein abundance changes.

Here we have developed a chemical proteomics method integrating protein labeling, click chemistry and multiplexed proteomics, which can effectively overcome the challenges with existing methods used to study protein dynamics. Newly synthesized proteins were selectively enriched and their abundance changes were quantified as a function of time. We studied protein dynamics in the cell cycle because it is highly dynamic and well-regulated, and is one of the most important events in biological systems.^14^ The S phase, *i.e.* the synthesis phase, is a critical stage of the cell cycle during which DNA is replicated. Over 1400 newly synthesized proteins were identified in the S phase in HepG2 cells, including three cyclins as expected. These newly synthesized proteins were selectively enriched at multiple time points, and their abundance changes were quantified. The half-lives of many newly synthesized proteins were accurately obtained. This method can be extensively applied to investigate protein dynamics in biological systems.

## Results and discussion

### The principle of the selective enrichment of newly synthesized proteins in the S phase

**Fig. 1:**
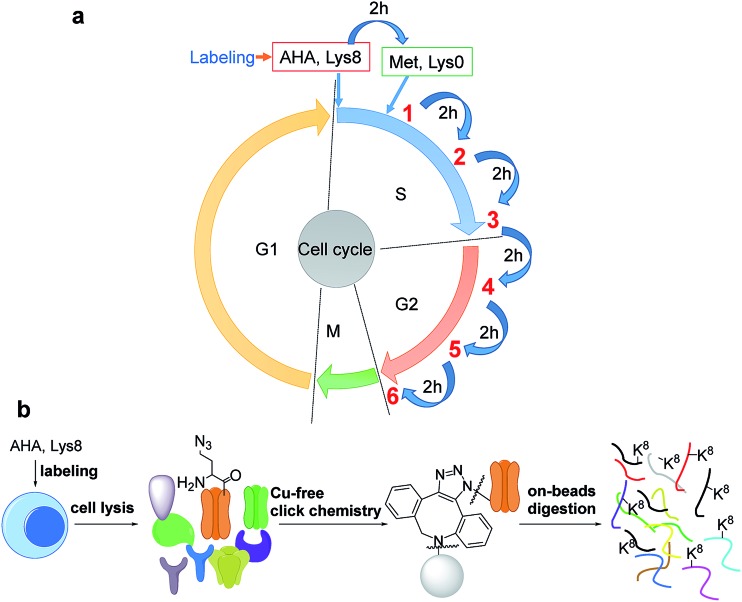
Experimental principle and procedure: (a) cells were labeled with AHA and heavy lysine. After cells were labeled by AHA for two hours, normal medium containing methionine and light lysine were used to culture cells; cells were harvested every two hours; (b) copper-free click chemistry was employed to selectively enrich newly synthesized proteins in the S phase.

The introduction of bio-orthogonal groups into proteins has provided an effective method that incorporates a chemical handle for the investigation of protein dynamics and functions.^15^–^19^ Several groups have been incorporated into proteins, including azido and alkyne groups.^18^,^20^–^27^ Azidohomoalanine (AHA), an analog of methionine, was employed to effectively label proteins with an azido group by replacing methionine during protein synthesis.^28^,^29^ Here, cells were first synchronized in the early S phase by the double thymidine block method,^30^ and then double labeled with heavy lysine (+8 D) and AHA, as shown in Fig. 1a. As a result, newly synthesized proteins were labeled by AHA and heavy lysine. AHA labeling enabled the selective enrichment of newly synthesized proteins, while labeling proteins with heavy lysine allowed us to readily distinguish newly synthesized proteins from non-specifically bound existing proteins.

Newly synthesized proteins labeled with AHA were separated and enriched using magnetic beads conjugated with dibenzocyclooctyne (DBCO) (Fig. 1b).^19^,^31^ The copper-free click reaction between DBCO and the azido group incorporated into proteins is quick, efficient, and does not require any heavy metal ions that may damage the protein backbone. After proteins containing the azido group were covalently bound to beads, stringent washes were performed to remove non-specifically bound proteins. Finally, on-beads digestion was carried out and the resulting peptides were detected by LC-MS/MS.

### Identification of newly synthesized proteins in the S phase

During the S phase of the cell cycle, cells prepare for their division in the M phase and their genome is doubled. Each of two functional daughter cells requires not only a complete copy of DNA, but also many essential proteins for survival. More importantly, protein abundance changes are critical for cells to regulate the cell cycle, and a well-known example is the oscillation of cyclin abundances throughout the cell cycle. Although there has been a long-standing interest to determine which proteins are synthesized in the S phase, it is extraordinarily challenging to selectively analyze them for the following reasons. First, newly synthesized proteins are often present at low abundance, which are frequently masked by many high-abundance existing proteins. Second, here we are only interested in the dynamics of newly synthesized proteins, which need to be clearly distinguished from existing proteins in the cell. Therefore, effective separation and tagging are essential to analyze these newly synthesized proteins prior to MS analysis.

**Fig. 2:**
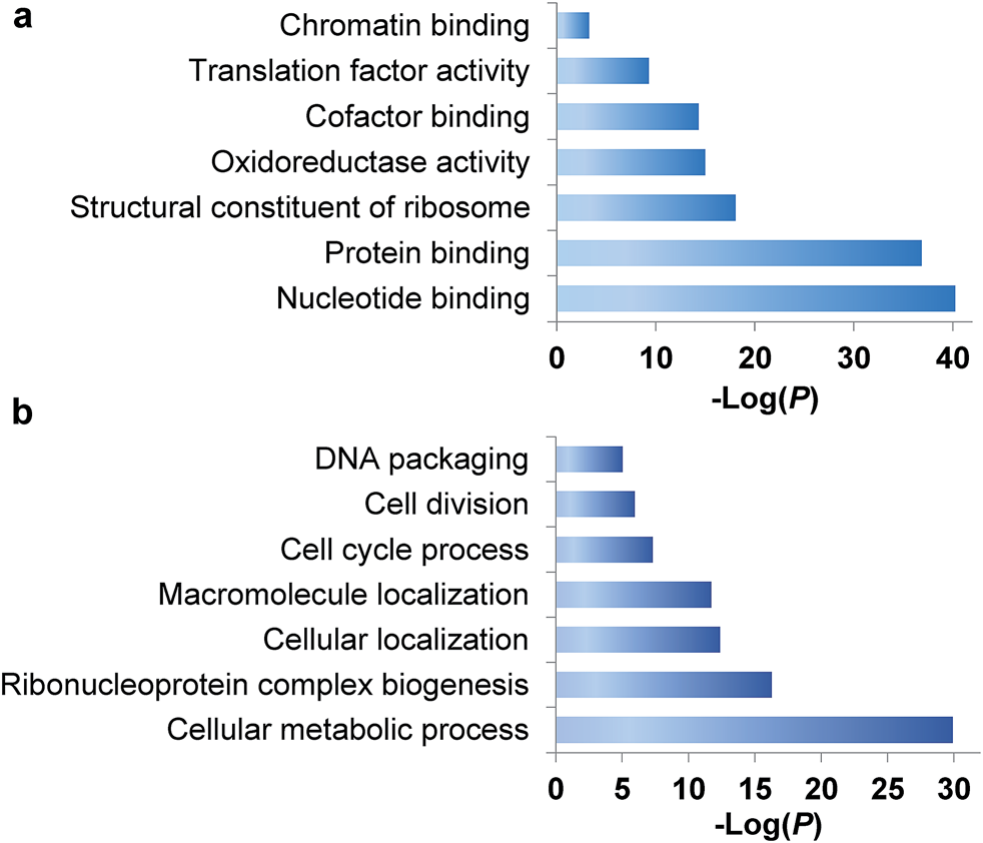
Clustering of newly synthesized proteins identified in the S phase based on (a) molecular function and (b) biological process.

A total of 1426 newly synthesized proteins were identified in the S phase (Table S1†). The newly synthesized proteins in the S phase were clustered based on Gene Ontology (GO) analysis from the Database for Annotation, Visualization and Integrated Discovery (DAVID)^32^ and the Protein ANalysis THrough Evolutionary Relationships (PANTHER) classification system,^33^,^34^ and the results are shown in Fig. 2. Based on molecular function, proteins with nucleotide binding functions were the most highly enriched with a very low *P* value of 5.6 × 10^–41^, and those with protein binding functions were also very highly enriched (*P* = 1.4 × 10^–37^). It is anticipated that during DNA duplication, many proteins related to nucleotide and protein binding are synthesized and active, including helicases, topoisomerases, DNA polymerases and transcription factors. Structural constituents of the ribosome were highly enriched as well because in addition to DNA, many essential proteins are required to double before the M phase, including the ribosome itself.

Based on biological process, proteins related to cellular metabolic process were the most highly enriched, and 56 newly synthesized proteins corresponded to ribonucleoprotein complex biogenesis (*P* = 5.2 × 10^–17^). This is consistent with the enriched category of structural constituents of the ribosome discussed above. There were 90 newly synthesized proteins corresponding to the cell cycle process, and 53 proteins related to cell division (*P* = 1.1 × 10^–6^). Protein clustering based on cellular component revealed that proteins in both the ribosome and splicesome were highly enriched with *P* values of 2.7 × 10^–23^ and 2.5 × 10^–10^, respectively. These results clearly demonstrated that in addition to DNA duplication, protein synthesis was also highly active in the early stage of the S phase.

### Cyclins and transcription factors identified in the S phase

Cyclins are a family of proteins that control the progression of the cell cycle by activating cyclin-dependent kinase (CDK) enzymes, and their concentration varies in a cyclical fashion during the cell cycle. This group of proteins has been extensively studied and information regarding their abundance oscillation throughout the cell cycle has been well-documented,^35^–^38^ therefore the identification of cyclins in the S phase can be used as an excellent standard to examine the effectiveness of the method.

Cyclin D forms complexes with CDK4 or CDK6, which play key roles in the G1/S transition; the synthesis of cyclin D is initiated during G1. Cyclin E binds to CDK2 in the G1 phase, which is required for the transition from the G1 to S phase.^39^ The concentrations of cyclins D and E reach their peaks in the transition of G1/S.^40^ As expected, newly synthesized cyclins D and E were not identified in the S phase.

Cyclins A and B are present at the lowest concentrations in the early S phase, based on reports in the literature.^39^,^41^ Cyclin A has two distinct isoforms: A1 – the embryonic-specific form, and A2 – the somatic form. Cyclin A1 is mainly expressed during meiosis and in early stages of embryogenesis, while cyclin A2 is expressed in dividing somatic cells,^42^ including HepG2 cells. Therefore, only cyclin A2 was identified in this work. Cyclin B interacts with CDK1, and is necessary for the progression of cells into and out of the M phase. The amount of cyclin B and the activity of the cyclin B–CDK1 complex increase until mitosis, where they decline dramatically due to degradation of cyclin B.^43^ The synthesis of cyclin B starts in the early S phase. As expected, two cyclin B isoforms, B1 and B2, were identified. Cyclin B3 was not identified, as it is a testis specific cyclin expressed in developing germ cells in the testis, but weakly or not expressed in other tissues.^44^,^45^

Previously siRNAs and time-lapse epifluorescence microscopy were employed to examine the roles of various candidate mitotic cyclins in chromatin condensation in HeLa cells.^41^ Cyclin A2 helps initiate mitosis, and cyclins B1 and B2 are particularly critical for the maintenance of the mitotic state.^41^ In this work, we identified only cyclins A2, B1 and B2, which correspond extremely well with reports in the literature,^46^ considering the cells used and the newly synthesized proteins expected to be in the early S phase. The identification of these three cyclins firmly indicated that the enrichment is highly effective to selectively enrich newly synthesized proteins.

Transcription factors are another critical group of proteins; they regulate gene expression in cells but are normally present at very low abundance. It is extremely challenging to detect them by using discovery-based proteomics techniques because they are often buried by many high-abundance proteins. Among 1426 newly synthesized proteins identified in the S phase, 9.6% of them were transcription factors (137 proteins, Table S2†), which is slightly greater than the normal distribution of transcription factors (∼8% of total genes). This further demonstrated that the current methods can effectively enrich low-abundance newly synthesized proteins.

### Quantify the abundance changes of newly synthesized proteins

**Fig. 3:**
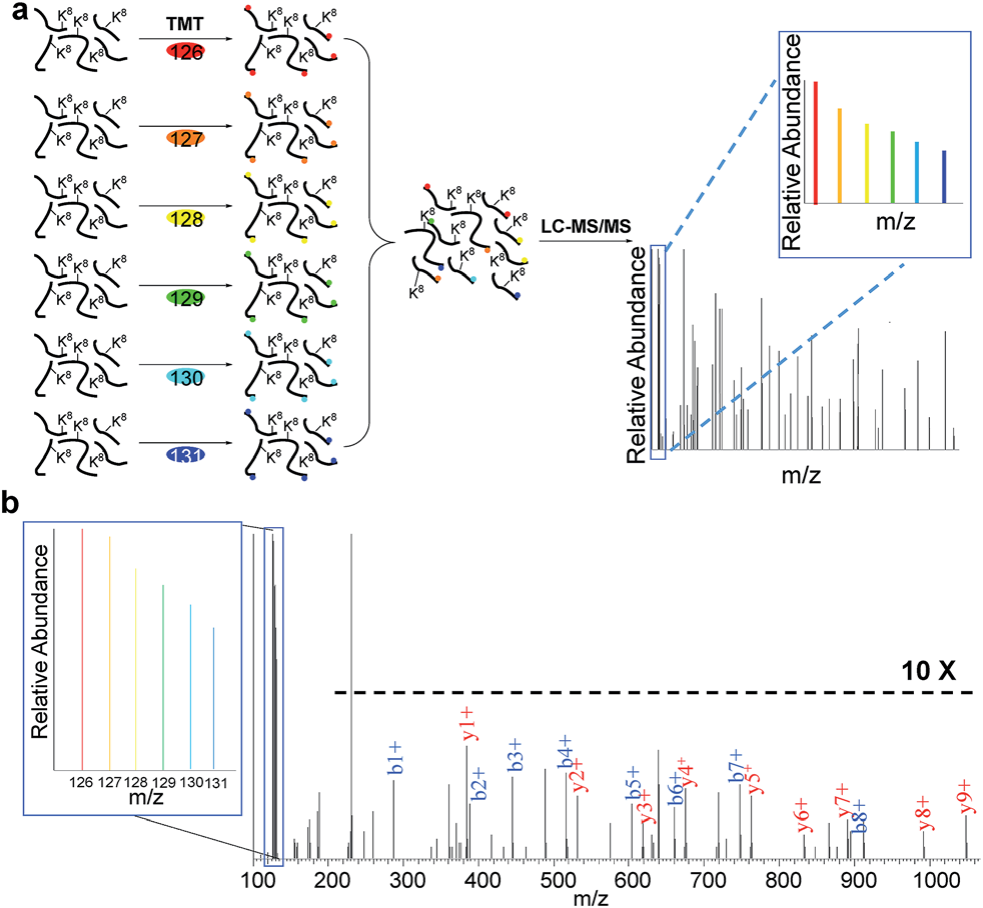
Measurement of half-lives of newly synthesized proteins in the S phase by combining copper-free click chemistry enrichment and TMT labeling: (a) experimental procedure for quantification of proteins; (b) example mass spectrum showing protein identification and abundance changes.

In eukaryotic cells, protein degradation is mainly mediated by two pathways: the ubiquitin–proteasome and lysosomal proteolysis. The half-lives vary greatly, and differing rates of protein degradation are related to the regulation of cellular events. Therefore, accurate measurement of protein abundance changes and their half-lives will provide insight into protein properties and functions, and cellular activities. Here we specifically quantified the abundance changes of these newly synthesized proteins in living cells, and measured their half-lives. After newly synthesized proteins were labeled by AHA and heavy lysine during the early S phase, cells were chased with regular medium containing nocodazole to prevent them from entering the M phase, and subsequently harvested at six time points (0, 2, 4, 6, 8 and 10 hours), as shown in Fig. 3. Similar to the procedure above, newly synthesized proteins bearing the azido group in the S phase were selectively separated and enriched using click chemistry. After digestion of enriched proteins, peptides from each of the six samples were labeled with different tandem mass tag (TMT) reagents (Fig. 3a). All enriched samples were mixed based on the number of cells harvested at each time point. As a result, the half-lives were not affected by protein dilution resulting from cell growth. The fragments in the tandem mass spectra enabled us to confidently identify peptides, and reporter ion intensities were used to quantify peptide and corresponding protein abundance changes. One example is shown in Fig. 3b; based on the fragmentation, we were able to confidently identify the peptide YDVENC*LANK# (* refers to the alkylation of cysteine and # indicates heavy lysine). The identified peptide is from MCM3, which is a DNA replication licensing factor. The abundance changes were measured from the reporter ion intensities, as shown in Fig. 3b. The protein half-lives were calculated based on the protein abundance changes with the most commonly used exponential decay equation:^10^

1 *P* = *P*_0_e^–*kt*^

where *P*_0_ is the intensity of the reporter ion at the first time point, *P* is the intensity of the reporter ion at each subsequent time point, *k* is the degradation rate constant and *t* is time. Therefore, the following equation shows the ratio of the intensities:

2 *P*/*P*_0_ = e^–*kt*^

The ratios at the six time points allowed us to calculate the half-life (*t*_1/2_) of each protein, *i.e.* the time point when only half of the newly synthesized protein remain in cells. After strict data filtering, the half-lives of 803 newly synthesized proteins in the S phase were obtained (listed in Table S3†).

The quantification of peptides based on reporter ion intensities, such as with the TMT method, could result in ratio suppression due to the potential interferences from other ions.^47^ However, here it should not be a problem because of several reasons. First, compared to whole cell lysates, the enriched samples were much simpler because only newly synthesized proteins within the first two hours of the early S phase were selectively separated, which is consistent with the identification of only 1400 proteins compared to ∼10 000 proteins that are typically expressed in cells. Second, fractionating enriched peptides with HPLC made each of the twenty fractions much simpler. In addition, a long gradient in the LC-MS/MS method allowed peptides to be further separated. In our experiment, the combination of higher-energy collisional dissociation (HCD) and fragment detection, including reporter ions, in the high mass accuracy and high resolution Orbitrap cell allowed us to confidently identify and accurately quantify peptides. The ratios of the reporter ions were further calibrated based on the isotopic distribution. If ratio suppression existed, the calculated half-lives would be relatively long. However, the values here are shorter than those obtained with the SILAC pulse-chase method,^10^,^13^ and are in very good agreement with the values obtained using a MS-independent method.^48^

### Distribution of protein half-lives

**Fig. 4:**
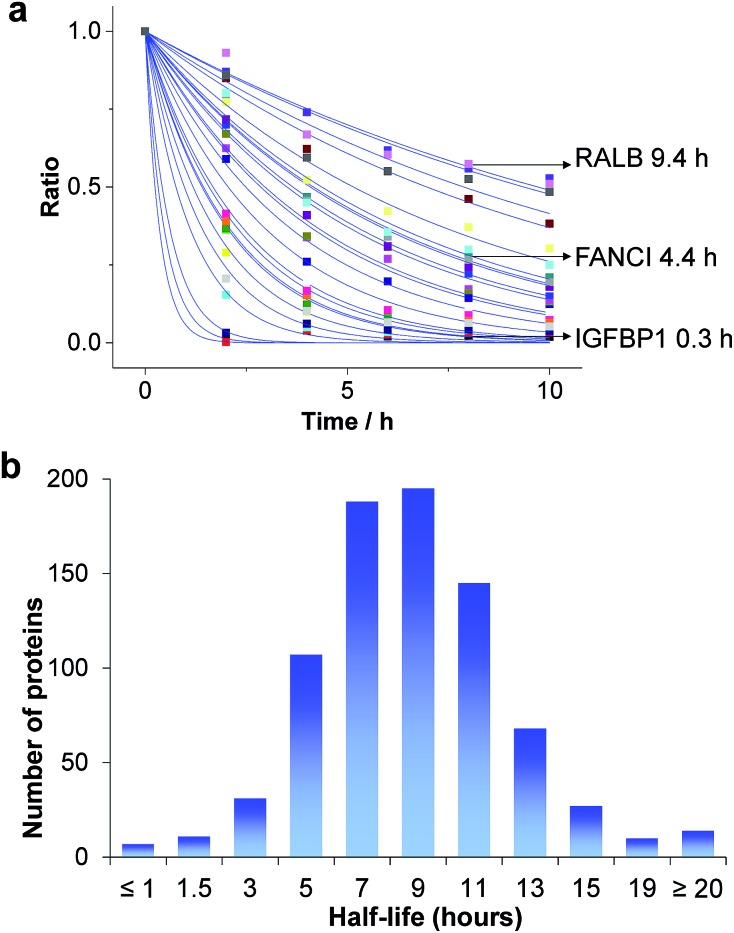
Half-lives of newly synthesized proteins in the S phase: (a) abundance changes of several proteins and the simulation to determine their half-lives; (b) distribution of protein half-lives.

Based on the protein abundance changes measured through the multiplexed proteomics experiment, we can confidently calculate the half-lives of 803 proteins. The abundance changes of several proteins and their half-life traces based on eqn (2) are shown in Fig. 4a. For example, RALB is a GTPase, and is involved in a variety of cellular processes including gene expression, cell migration and proliferation, and membrane trafficking. The half-life was measured to be 9.4 hours in this work. IGFBP1 is insulin-like growth factor-binding protein 1, which is a well-known secreted protein. It has a very short half-life of 0.3 hours inside the cell because it is secreted after synthesis. The distribution of all protein half-lives is shown in Fig. 4b. The bin at 9 hours (half-lives between 8 and 10 h) contains the greatest number of proteins. The majority of the proteins quantified have half-lives within the range of 4–14 hours. About 6% of all quantified proteins (49) have half-lives <4 hours, while 51 proteins have long half-lives (>14 hours); the median half-life is 8.7 hours.

**Fig. 5:**
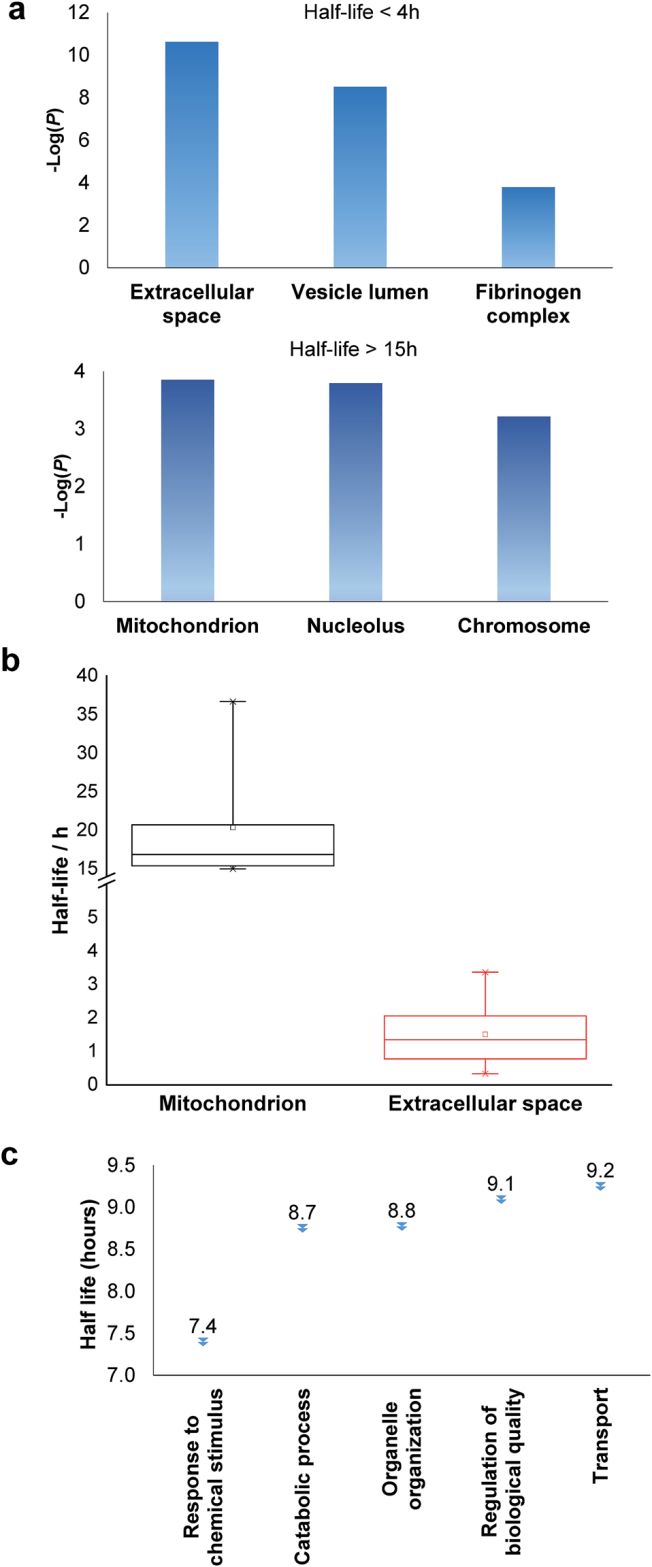
Clustering of proteins with different half-lives: (a) enriched proteins among those with short and long half-lives; (b) comparison of the median half-lives among proteins located in mitochondrion and extracellular space (the half-lives of proteins in the extracellular space refer to the intracellular half-lives); (c) the median half-lives for proteins with different functions.

Among the proteins with a short half-life in cells, those secreted to the extracellular region were found to be the most highly enriched. For example, F5 (coagulation factor *V*) has a half-life of 0.8 hours, and APOB has a half-life of 1.3 hours. These values clearly demonstrate that the method worked well because secreted proteins are known to be translocated to the extracellular space shortly after synthesis. Proteins in the vesicle lumen and the fibrinogen complex are also short-lived in cells (Fig. 5a). The fibrinogen complex is highly soluble, and found in blood plasma and involved in clot formation, which is located outside of the cell.

Among long-lived proteins (half-lives > 15 hours), the top three enriched groups of proteins were located in the mitochondrion, nucleolus and chromosome (Fig. 5a). Normally proteins in the mitochondrion are relatively more stable, and 11 long-lived proteins were located in the mitochondrion. Histones are another example of typically long-lived proteins, and here, several histones were identified with half-lives over 10 hours. For example, based on 7 unique and 17 total quantified peptides, the half-life of HIST1H1B was calculated to be 15.7 hours. Previously the half-life of another histone (H2AFV) was calculated to be 13.9 hours using a MS-independent method.^48^ As shown in Fig. 5b, the median half-life of proteins located in the mitochondrion is 16.9 hours. In striking contrast, the median half-life of proteins in the extracellular space is over ten times shorter (only 1.3 hours). Several categories of proteins with distinct functions are shown in Fig. 5c. Proteins corresponding to chemical stimuli have the shortest median half-life, while proteins with transportation functions are the longest-lived among these five categories. Eukaryotic initiation factors (eIFs) are proteins involved in the initiation phase of eukaryotic translation, and they form a complex with the ribosomal subunit to regulate protein synthesis. Half-lives of a group of eukaryotic translation initiation factors were listed in Table 1. The median half-life is 7.1 hours and the majority of proteins have half-lives less than 8 hours.

**Table 1:** Examples of half-lives measured for eukaryotic translation initiation factors

Gene symbol	UniProt ID	Protein half-life		Annotation
		This work	Previous work^48^	
EIF1AX	P47813	7.7		Eukaryotic translation initiation factor 1A
EIF2S2	P20042	6.3	8.3	Eukaryotic translation initiation factor 2 subunit 2
EIF3E	P60228	8.3		Eukaryotic translation initiation factor 3 subunit E (eIF3e)
EIF3H	O15372	7.0		Eukaryotic translation initiation factor 3 subunit H (eIF3h)
EIF3L	Q9Y262	12.2		Eukaryotic translation initiation factor 3 subunit L (eIF3l)
EIF4A1	P60842	6.0	7.3	Eukaryotic initiation factor 4A-I
EIF4A2	Q14240	5.7		Eukaryotic initiation factor 4A-II
EIF4A3	P38919	6.4		Eukaryotic initiation factor 4A-III
EIF4B	P23588	7.4		Eukaryotic translation initiation factor 4B (eIF-4B)
EIF4G1	Q04637	6.8		Eukaryotic translation initiation factor 4 gamma 1
EIF4G2	P78344	9.6		Eukaryotic translation initiation factor 4 gamma 2
EIF5B	O60841	8.2	8.9	Eukaryotic translation initiation factor 5B

There have already been several reports regarding the measurement of protein half-lives by using SILAC-based pulse-chase combined with MS-based proteomics.^10^,^13^ The current results are quite different from those obtained from the SILAC methods. In pulse-chase SILAC experiments, heavy amino acid recycling is an inherent problem. By using the current method, AHA and heavy lysine can still be recycled, but the possibility of recycling both AHA and heavy lysine in a single identified peptide is extremely low. Furthermore, the protein half-lives obtained from the pulse-chase SILAC experiments were based on the ratios measured independently by MS, and errors are more likely as a result. Here we quantified proteins at six time points, and the peptide abundance changes at these time points were measured in a single MS^2^ spectrum. The half-lives of proteins calculated from accurately measured protein abundance changes at six points are expected to be more reliable. More importantly, in our work, we can selectively enrich those newly synthesized proteins, which is beyond the reach of normal pulse-chase SILAC experiments. One drawback of AHA labeling is that AHA could affect protein folding and stability, but the comparison of our results with those reported using a MS-independent method suggests that this is less likely to be true. Protein half-lives reported here are very similar to those measured by a MS-independent method, *i.e.* the bleach-chase experiment with fluorescence detection.^48^ For example, the half-lives of EIF2S2, EIF4A1 and EIF5B were calculated to be 6.3, 6.0 and 8.2 hours in this work, respectively, and they were reported to be 8.3, 7.3 and 8.9 hours with the bleach-chase method in literature.^48^ Overall, the median half-life of 100 proteins measured in the bleach-chase experiment was 8.2 hours,^48^ which is in excellent agreement with the median half-life of 8.7 hours for 803 newly synthesized proteins in the S phase calculated here.

## Conclusions

The cell cycle is an extremely complex and well-regulated event that is essential for life. Multiple factors contribute to the regulation of the cell cycle, including protein abundance changes, modifications and interactions. Many aspects remain unknown, including which proteins are synthesized in each stage of the cell cycle and the dynamics of these proteins. In this work, a novel chemical proteomics method integrating bio-orthogonal amino acid labeling and copper-free click chemistry was developed to selectively separate newly synthesized proteins during the early S phase of the cell cycle. More than 1400 newly synthesized proteins were identified in the S phase, including cyclin A2, B1 and B2, which is very consistent with reports in the literature. Because this method selectively targets newly synthesized proteins, it enabled us to identify low-abundance proteins, including 137 transcription factors. By combining this method with multiplex quantitative proteomics techniques, the abundance changes of these newly synthesized proteins in the S phase were quantified after being selectively separated at six different time points and their half-lives were calculated. The median half-life of over 800 quantified proteins was 8.7 hours, which corresponds very well with the median half-life of 8.2 hours for 100 proteins in previous research. Proteins with shorter half-lives in cells are typically secreted, while those in the mitochondrion and nucleus are long-lived. The identification of newly synthesized proteins in the early S phase and the measurement of their half-lives provide very useful information to further understand the molecular mechanisms of the cell cycle, and protein properties and functions.

Although bio-orthogonal amino acid labeling, click chemistry and TMT tagging are well-documented, here we have designed and tailored a novel method integrating these approaches to globally study protein dynamics and measure their half-lives, which overcomes problems associated with existing methods. The current method has several advantages. *First*, double labeling with AHA and heavy lysine allows us to selectively enrich newly synthesized proteins and clearly distinguish them from existing proteins. *Second*, double labeling can significantly reduce the impact of heavy amino acid recycling on the measurement of protein abundance changes in the pulse-chase SILAC experiments. *Third*, interferences from protein dilution due to cell growth is eliminated because cells were arrested against cell division, and newly synthesized protein abundance changes are based on the same number of cells, not amount of proteins. *Fourth*, multiplexed proteomics enabled us to quantify proteins at several time points simultaneously, therefore increasing the accuracy of measuring protein abundance changes and the corresponding half-lives. *Fifth*, the high throughput MS-based experiment enabled us to study protein dynamics on a global level, instead of a one-to-one fashion. *Lastly*, over eight hundred protein half-lives were accurately measured, which provides valuable information to the biological community. This method can be extensively applied to investigate newly synthesized proteins and their dynamics in the biological and biomedical research fields.

## Supplementary Material

Supplementary information

Supplementary information

Supplementary information

Supplementary information
