# Supplementary material for: Systematic study of the dynamics and half-lives of newly synthesized proteins in human cells
Source: Chem Sci. 2015 Nov 16;7(2):1393–400. doi: 10.1039/c5sc03826j (PMC5975921; doi:10.1039/c5sc03826j)
Supplement: Supplementary file 1 [file SC-007-C5SC03826J-s001.pdf]

Electronic Supplementary Material (ESI) for Chemical Science.
This journal is © The Royal Society of Chemistry 2015

Supplementary Information

Systematic Study of the Dynamics and Half-lives of Newly Synthesized Proteins in Human Cells

*Weixuan Chen, Johanna M. Smeekens and Ronghu Wu**

School of Chemistry and Biochemistry and the Petit Institute for Bioengineering and Bioscience, Georgia Institute of Technology, Atlanta, Georgia 30332, USA

* Corresponding author: Phone: 404-385-1515; Fax: 404-894-7452.

E-mail: ronghu.wu@chemistry.gatech.edu

Methods

Materials and bead derivatization. Complete protease inhibitors were purchased from Roche Applied Sciences, and sequencing grade Lysyl endopeptidase (Lys-C) from Wako. Thymidine, nocodazole, propidium iodide (PI), Dulbecco's Modified Eagle Medium (DMEM), and phosphate buffered saline (PBS) were from Sigma-Aldrich. Dialyzed fetal bovine serum (diFBS), amine-reactive 6-plex tandem mass tag (TMT) reagents, and amine derivatized MagnaBind beads were purchased from Fisher Scientific. L-azidohomoalanine (AHA) and dibenzocyclooctyl (DBCO)-NHS were from Click Chemistry Tools. DBCO modified magnetic beads were synthesized by mixing amine derivatized beads with DBCO-NHS in DMSO overnight and washing with PBS.

Cell lines, cell culture, cell cycle arrest, and metabolic labeling. Hep G2 cells were purchased from ATCC and cultured per the provided instructions. A double thymidine block was performed as previously described[1] and cells were synchronized at the G1/S phase transition. Medium was replaced with 2 mM thymidine in DMEM depleted of Lys and Met during the last 30 minutes of synchronization. Then cells depleted of Lys and Met were incubated with DMEM containing 10% diFBS, 0.8 mM Lys8 and 0.1 mM AHA for 2 hours. After AHA labeling during the early S phase, cells were chased by regular DMEM (containing nocodazole) and harvested at six different time points: 0, 2, 4, 6, 8 and 10 hours.

Cell lysis, protein extraction, newly synthesized protein enrichment and digestion. The cell pellets were washed in ice-cold PBS twice, suspended in ice-cold RIPA buffer containing 100 mM 4-(2-hydroxyethyl)-1-piperazineethanesulfonic acid (HEPES), pH=7.9, 150 mM NaCl, 0.5 % sodium dodecyl sulfate (SDS), benzonase (1 U/mL), and protease inhibitor mixture (Roche Diagnostics) and the suspension was incubated with shaking for 1 hour at 4 °C. After complete solubilization of nuclei and digestion of genomic DNA, the lysate was centrifuged at 25,000 *g* for 20 minutes. The supernatant was collected and newly synthesized proteins were enriched through incubation with DBCO derivatized beads at 4 °C overnight. The next day non-enriched proteins were removed from the magnetic beads by sequential washes: 10 washes with the same volume (100 µL) of RIPA buffer containing 2.5 % SDS and 2.5 % sodium deoxycholate (SDC), followed by another 10 washes with the same volume (100 µL) of 100 mM HEPES buffer containing 8 M urea, 10 % isopropanol, and 10 % acetonitrile. After stringent washing, proteins bound to the magnetic beads were reduced by 5 mM dithiothreitol (DTT) (56 °C, 25 minutes) and alkylated with 15 mM iodoacetamide (RT, 30 minutes in the dark). The magnetic beads with enriched newly synthesized proteins were resuspended in 50 mM HEPES (pH=8.2), 0.1 M urea, 5 % ACN, digested with Lys-C at 31°C for 15 hours. Digestion was quenched by the addition of 10 % TFA to a final concentration of 0.4 %, the supernatant was collected and the beads were washed with 0.4 % TFA twice. Each peptide sample was purified using a Sep-Pak tC18 cartridge.

Peptide TMT labeling, fractionation and LC-MS/MS analysis. Purified and dried peptide samples from each time point were tagged with TMT reagents according to the vendor's protocol. The six labeled peptide samples were combined and desalted again using a tC18 Sep-Pak cartridge. Then peptides were fractionated using high-pH reversed phase high performance liquid

chromatography (HPLC) (pH=10). The sample was separated into 20 fractions using a 4.6 × 250 mm 5µm particle reversed phase column (Waters) with a 40 minute gradient of 5-30% acetonitrile (ACN) containing 25 mM ammonium acetate. The TMT labeled fractionated peptide samples were dried and then dissolved in a solvent containing 5% ACN and 4% formic acid (FA), and 4 µL was loaded onto a microcapillary column packed with C18 beads (Magic C18AQ, 5 µm, 200 Å, 100 µm x 16 cm) using a WPS-3000TPLRS autosampler (UltiMate 3000 Thermostatted Pulled Loop Rapid Separation Wellplate Sampler, Dionex). Peptides were separated by reversed-phase chromatography using an UltiMate 3000 binary pump with a 90-minute gradient of 4-30% ACN (in 0.125% FA) and detected in a hybrid dual-cell quadrupole linear ion trap – Orbitrap mass spectrometer (LTQ Orbitrap Elite, ThermoFisher, with Xcalibur 3.0.63 software) using a data-dependent Top 15 method. For each cycle, one full MS scan (resolution: 60,000) in the Orbitrap at 10^6 AGC target was followed by up to 15 MS/MS for the most intense ions. Selected ions were excluded from further analysis for 90 s each. Ions with a single or unassigned charge were not sequenced. MS/MS scans were activated by HCD at 40.0% normalized collision energy with 1.2 m/z isolation width, and detected in the orbitrap cell.

Database searching, data filtering, and quantification. The raw files recorded by MS were converted into mzXML format. Precursors for MS/MS fragmentation were checked for incorrect monoisotopic peak assignments while refining precursor ion mass measurements[2]. All MS/MS spectra were then searched using the SEQUEST algorithm (version 28)[3]. Spectra were matched against a database encompassing sequences of all proteins in the Uniprot Human (*Homo sapiens*) database (downloaded in February 2014) and common contaminants such as keratins. Each protein sequence was listed in both forward and reversed orientations to estimate the false

discovery rate (FDR) of peptide identifications. The following parameters were used for the database search: 20 ppm precursor mass tolerance; 1.0 Da product ion mass tolerance; full Lys-C digestion; up to two missed cleavages; variable modifications: oxidation of methionine (+15.9949), lysine (+8.0142); fixed modifications: carbamidomethylation of cysteine (+57.0214), N-terminus TMT modification (+229.1629).

The target-decoy method was employed to evaluate and further control FDRs of peptide identification[4, 5]. Linear discriminant analysis (LDA) was utilized to distinguish correct and incorrect peptide identifications using numerous parameters such as XCorr, ΔCn, and precursor mass error[2, 6, 7]. This approach has been used in previous work[8-11]. After scoring, peptides less than six amino acids in length were discarded and peptide spectral matches were filtered to a less than 1% FDR based on the number of decoy sequences in the final data set.

The TMT reporter ion intensities in the tandem mass spectra were employed to quantify confidently identified peptides. The isotopic information provided by the company (Thermo) was used to calibrate the measured intensities. The median intensity ratio for each unique peptide in each channel was obtained, and eventually the median value of all unique peptides for each corresponding protein was calculated as the protein ratio.

Bioinformatic analysis. Protein annotations were extracted from the UniProt database (http://www.uniprot.org/), and transcription factors were based on literature[12]. Protein function annotations were obtained using DAVID (http://david.abcc.ncifcrf.gov/home.jsp)[13] and the Protein ANalysis THrough Evolutionary Relationships (PANTHER) classification system[14, 15].

References:

1. J. Harper, in *Cell Cycle Control*, eds. T. Humphrey and G. Brooks, Humana Press, 2005, vol. 296, ch. 7, pp. 157-166.
2. E. L. Huttlin, M. P. Jedrychowski, J. E. Elias, T. Goswami, R. Rad, S. A. Beausolei, J. Villen, W. Haas, M. E. Sowa and S. P. Gygi, *Cell*, 2010, 143, 1174-1189.
3. J. K. Eng, A. L. McCormack and J. R. Yates, *J. Am. Soc. Mass Spectrom.*, 1994, 5, 976-989.
4. J. M. Peng, J. E. Elias, C. C. Thoreen, L. J. Licklider and S. P. Gygi, *J Proteome Res*, 2003, 2, 43-50.
5. J. E. Elias and S. P. Gygi, *Nat Methods*, 2007, 4, 207-214.
6. W. X. Chen, J. M. Smeekens and R. H. Wu, *J Proteome Res*, 2014, 13, 1466-1473.
7. W. X. Chen, J. M. Smeekens and R. H. Wu, *Mol Cell Proteomics*, 2014, 13, 1563-1572.
8. X. Du, S. J. Callister, N. P. Manes, J. N. Adkins, R. A. Alexandridis, X. Zeng, J. H. Roh, W. E. Smith, T. J. Donohue, S. Kaplan, R. D. Smith and M. S. Lipton, *J Proteome Res*, 2008, 7, 2595-2604.
9. L. Kall, J. D. Canterbury, J. Weston, W. S. Noble and M. J. MacCoss, *Nat Methods*, 2007, 4, 923-925.
10. J. Y. Zhang, J. Ma, L. Dou, S. F. Wu, X. H. Qian, H. W. Xie, Y. P. Zhu and F. C. He, *Mol Cell Proteomics*, 2009, 8, 547-557.
11. R. H. Wu, W. Haas, N. Dephoure, E. L. Huttlin, B. Zhai, M. E. Sowa and S. P. Gygi, *Nat Methods*, 2011, 8, 677-U111.
12. T. Ravasi, H. Suzuki, C. V. Cannistraci, S. Katayama, V. B. Bajic, K. Tan, A. Akalin, S. Schmeier, M. Kanamori-Katayama, N. Bertin, P. Carninci, C. O. Daub, A. R. R. Forrest, J. Gough, S. Grimmond, J. H. Han, T. Hashimoto, W. Hide, O. Hofmann, H. Kawaji, A. Kubosaki, T. Lassmann, E. van Nimwegen, C. Ogawa, R. D. Teasdale, J. Tegner, B. Lenhard, S. A. Teichmann, T. Arakawa, N. Ninomiya, K. Murakami, M. Tagami, S. Fukuda, K. Imamura, C. Kai, R. Ishihara, Y. Kitazume, J. Kawai, D. A. Hume, T. Ideker and Y. Hayashizaki, *Cell*, 2010, 140, 744-752.
13. D. W. Huang, B. T. Sherman and R. A. Lempicki, *Nat Protoc*, 2009, 4, 44-57.
14. H. Y. Mi, B. Lazareva-Ulitsky, R. Loo, A. Kejariwal, J. Vandergriff, S. Rabkin, N. Guo, A. Muruganujan, O. Doremieux, M. J. Campbell, H. Kitano and P. D. Thomas, *Nucleic Acids Res.*, 2005, 33, D284-D288.
15. P. D. Thomas, M. J. Campbell, A. Kejariwal, H. Y. Mi, B. Karlak, R. Daverman, K. Diemer, A. Muruganujan and A. Narechania, *Genome Res.*, 2003, 13, 2129-2141.
